# Supplementary material for: Diversity and dynamics of bacteria from iron-rich microbial mats and colonizers in the Mediterranean Sea (EMSO-Western Ligurian Sea Observatory): Focus on Zetaproteobacteria
Source: PLoS One. 2024 Jul 15;19(7):e0305626. doi: 10.1371/journal.pone.0305626 (PMC11249232; doi:10.1371/journal.pone.0305626)
Supplement: S1 Fig — Rarefaction curves for Bacteria for each triplicate of each sample. (PDF) [file pone.0305626.s001.pdf]

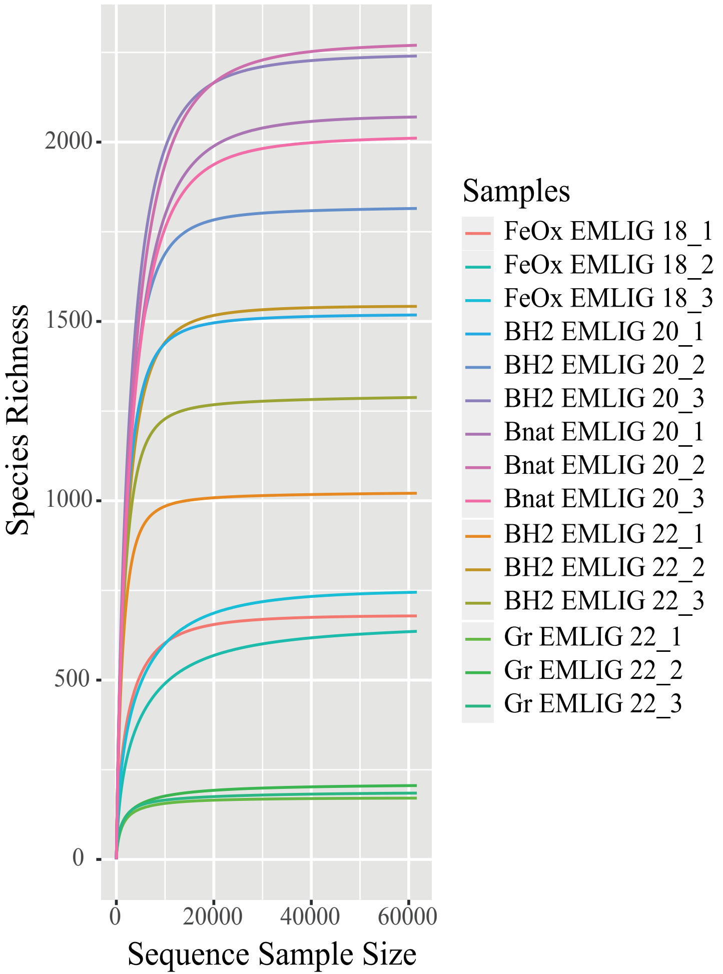

Species Richness
2000
1500
1000
500
0
0
20000
40000
60000
Sequence Sample Size
Samples
FeOx EMLIG 18_1
FeOx EMLIG 18_2
FeOx EMLIG 18_3
BH2 EMLIG 20_1
BH2 EMLIG 20_2
BH2 EMLIG 20_3
Bnat EMLIG 20_1
Bnat EMLIG 20_2
Bnat EMLIG 20_3
BH2 EMLIG 22_1
BH2 EMLIG 22_2
BH2 EMLIG 22_3
Gr EMLIG 22_1
Gr EMLIG 22_2
Gr EMLIG 22_3
