# Supplementary material for: Diversity and dynamics of bacteria from iron-rich microbial mats and colonizers in the Mediterranean Sea (EMSO-Western Ligurian Sea Observatory): Focus on Zetaproteobacteria
Source: PLoS One. 2024 Jul 15;19(7):e0305626. doi: 10.1371/journal.pone.0305626 (PMC11249232; doi:10.1371/journal.pone.0305626)
Supplement: S1 Table — Primers used for dPCR with respective annealing temperature and cycle number for denaturation step. (PDF) [file pone.0305626.s002.pdf]

Forward primer	Reverse Primer	Target DNA	Optimal annealing temp. dPCR	Number of denaturation cycle	Référence
Bac 1369F<br>5’-CGGTGAATACGTTCYCGG-3’	Prok 1492R<br>5’-GGWTACCTTGTTACGACTT-3’	V3-V4 regions 16S rRNA gene bacteria	60°C	75	Kato et al. 2009
Zeta 672F<br>5’-CGGAATTCCGTGTGTAGCAGT-3’	Zeta 837R<br>5’-GCCACWGYAGGGGTCCGATACC-3’	16S rRNA gene specific Zetaproteo bacteria	60°C	45	Laufer et al., 2015<br>Kato et al., 2009
