# Supplementary material for: Diversity and dynamics of bacteria from iron-rich microbial mats and colonizers in the Mediterranean Sea (EMSO-Western Ligurian Sea Observatory): Focus on Zetaproteobacteria
Source: PLoS One. 2024 Jul 15;19(7):e0305626. doi: 10.1371/journal.pone.0305626 (PMC11249232; doi:10.1371/journal.pone.0305626)
Supplement: S2 Table — Number of reads after each step of the DADA2 pipeline and percentage of retained reads after treatment. (PDF) [file pone.0305626.s003.pdf]

SAMPLES	INPUT	FILTERED	DENOISED	MERGED	NON CHIMERIC	FINAL RETAINED (%)
FeOx_EMLIG18_1	133288	105537	103687	96823	94321	70,76
FeOx_EMLIG18_2	161180	124061	120839	111628	105744	65,61
FeOx_EMLIG18_3	127641	100428	97774	88934	85397	66,90
BH2_EMLIG20_1	114642	85915	84428	77706	76597	66,81
BH2_EMLIG20_2	104562	78176	76484	67850	66669	63,76
BH2_EMLIG20_3	120445	89121	86469	73710	71981	59,76
Bnat_EMLIG20_1	126318	93842	90882	78218	76382	60,47
Bnat_EMLIG20_2	144000	107233	103881	90092	87742	60,93
Bnat_EMLIG20_3	108990	80009	76978	64623	62843	57,66
BH2_EMLIG22_1	110871	84517	83178	77479	76795	69,17
BH2_EMLIG22_2	124703	95324	93504	85840	84285	67,59
BH2_EMLIG22_3	123131	93084	91580	85091	83335	67,68
Gr_EMLIG22_1	119447	95278	94046	91318	85252	71,37
Gr_EMLIG22_2	112132	89727	88152	85541	83402	74,38
Gr_EMLIG22_3	144944	116807	115369	112238	102439	70,67
